# Multidisciplinary Management of Spinal Dural Arteriovenous Fistulas Using an Endovascular-First Treatment Strategy: A Nine-Year Single-Center Experience

**DOI:** 10.3390/neurolint18070137

**Published:** 2026-07-16

**Authors:** Ivan Vukašinović, Bojana Zivkovic, Zarko Nedeljkovic, Mirko Micovic, Masa Petrovic, Lidija Stanic, Aleksandra Nedeljkovic, Tijana Nastasovic, Mihailo Milićević, Vladimir Bascarevic

**Affiliations:** 1 Center for Radiology and MRI, University Clinical center of Serbia, Pasterova 2, 11000 Belgrade, Serbia; 2 Clinic for Neurosurgery, University Clinical center of Serbia, Koste Todorovica 4, 11000 Belgrade, Serbia; 3 Faculty of Medicine, University of Belgrade, Dr Subotica Starijeg 8, 11000 Belgrade, Serbia; 4 Institute for Cardiovascular Diseases “Dedinje”, Heroja Milana Tepica 1, 11000 Belgrade, Serbia; 5 Department of Anesthesiology and Resuscitation on Neurosurgery Clinic, University Clinical Center of Serbia, Koste Todorovica 4, 11000 Belgrade, Serbia

**Keywords:** spinal dural arteriovenous fistula, embolization, microsurgical disconnection, myelopathy, digital subtraction angiography, endovascular therapy, spinal vascular malformation

## Abstract

Background/Objectives: Spinal dural arteriovenous fistulas (sDAVFs) are the most common spinal vascular malformation and a treatable cause of progressive myelopathy, yet diagnosis and optimal management remain challenging. This study presents a nine-year institutional experience using a multidisciplinary treatment algorithm that prioritizes endovascular embolization, with surgery reserved for unsuccessful or contraindicated cases. Methods: We retrospectively analyzed 15 patients treated between 2015 and 2023, all diagnosed by MRI and confirmed by digital subtraction angiography. Endovascular embolization was attempted as the initial treatment modality in all patients using contemporary liquid embolic agents. Results: Three patients (20%) subsequently required surgical disconnection following unsuccessful or incomplete embolization. Lesions ranged from Th5 to L5, and most patients presented with varying degrees of motor deficits, gait disturbance, paresthesias, or sphincter dysfunction. Neurological improvement occurred in all but one patient, and no treatment-related complications were observed. Prior embolization attempts aided intraoperative localization in surgically treated cases, facilitating precise fistula identification. Conclusions: These findings demonstrate the feasibility and favorable outcomes of a multidisciplinary, stepwise treatment strategy in this single-center experience. Endovascular embolization served as the initial treatment modality, while surgical disconnection provided an effective complementary option in selected cases where embolization was unsuccessful or incomplete.

## 1. Introduction

Spinal dural arteriovenous fistulas (sDAVFs) are uncommon vascular lesions of the spinal cord; nevertheless, they constitute the most prevalent subtype of spinal vascular malformations, accounting for approximately 70–80% of reported cases [1]. These lesions are defined by an abnormal arteriovenous shunt between a radiculomeningeal artery and a radicular vein within the dura mater, most frequently occurring at the level of the thoracolumbar spine. sDAVFs predominantly affect middle-aged to elderly individuals and exhibit a marked male predominance [2]. Despite their relative rarity, sDAVFs are associated with substantial morbidity, as delayed diagnosis and treatment may result in progressive and potentially irreversible neurological impairment [3].

The underlying pathophysiological mechanism of sDAVFs is venous hypertension secondary to arterialization of the spinal venous system, resulting in chronic venous congestion, impaired perfusion, and ischemic myelopathy. Clinically, patients typically present with slowly progressive motor and sensory deficits, gait disturbance, and, in advanced stages, sphincter dysfunction. The insidious onset and nonspecific symptomatology frequently result in misdiagnosis, often as degenerative spinal disease or inflammatory myelopathies, thereby contributing to prolonged diagnostic delays and suboptimal functional outcomes [3,4].

Although the precise etiology of sDAVFs remains incompletely elucidated, accumulating evidence supports an acquired rather than congenital origin [4]. Proposed mechanisms include age-related degenerative changes in the dural vasculature, venous thrombosis, local inflammation, or prior trauma, all of which may predispose to pathological arteriovenous shunting. In contrast to intracranial dural arteriovenous fistulas, sDAVFs rarely present with hemorrhagic complications and instead follow a chronically progressive course, further complicating timely recognition.

Advances in neuroimaging have substantially improved the detection and characterization of sDAVFs, contributing to earlier diagnosis and more precise treatment planning. Magnetic resonance imaging (MRI) serves as the initial diagnostic modality in most cases and typically reveals longitudinally extensive T2-weighted hyperintensity within the spinal cord, reflecting venous congestion-related edema. Serpiginous intradural flow voids along the dorsal surface correspond to dilated perimedullary veins. Involvement of the conus medullaris is frequently observed regardless of fistula level, likely related to orthostatic venous flow dynamics and gravitational influences on spinal venous drainage [5,6,7]. Following gadolinium administration, patchy intramedullary and serpentine perimedullary enhancement may be present, reflecting chronic venous hypertension and blood–spinal cord barrier disruption [8].

However, despite these imaging hallmarks, digital subtraction angiography (DSA) remains the definitive diagnostic standard, allowing precise identification of the fistulous point, arterial feeders, and venous drainage patterns. Importantly, DSA also provides the opportunity for immediate therapeutic intervention when anatomically feasible [2].

Historically, surgical interruption of the intradural draining vein was considered the definitive treatment for sDAVFs and remains associated with high rates of durable occlusion. Surgical management directly addresses the underlying venous hypertension and offers excellent long-term outcomes when performed successfully. Nevertheless, open spinal surgery is inherently invasive and carries risks including cerebrospinal fluid leakage, wound infection, delayed healing, and potential neurological complications.

Advances in endovascular techniques have substantially broadened the therapeutic armamentarium for sDAVFs, establishing embolization as a less invasive alternative to microsurgical disconnection. Endovascular therapy is based on superselective catheterization of the feeding radiculomeningeal artery, followed by occlusion of the fistulous connection using liquid embolic agents (LEAs). Historically, cyanoacrylate-based adhesives such as n-butyl cyanoacrylate (n-BCA) were widely used; however, their rapid polymerization and adhesive properties limited injection control and increased procedural complexity. More recently, non-adhesive ethylene vinyl alcohol (EVOH)-based agents have become predominant, offering improved handling characteristics and more controlled penetration of the fistulous point and proximal draining vein.

Currently available EVOH-based LEAs include Onyx (Covidien, Irvine, CA, USA), Squid (Balt Extrusion, Montmorency, France), PHIL (MicroVention, Tustin, CA, USA, and newer systems such as Menox™ (Meril Life Sciences, Vapi, India), which have demonstrated broadly comparable angiographic occlusion rates. Treatment success is influenced primarily by lesion angioarchitecture, venous drainage patterns, and operator experience rather than exclusive selection of embolic material [9].

Despite these technological advances, reported success rates of endovascular treatment for sDAVFs remain variable, typically ranging from 60% to 80%. Limitations persist in cases with unfavorable vascular anatomy, including shared arterial supply with critical spinal arteries such as the anterior spinal artery, as well as in situations where incomplete penetration of the draining vein predisposes to fistula persistence or recanalization. Consequently, while endovascular embolization represents an attractive first-line option due to its minimally invasive nature and the ability to combine diagnosis and treatment in a single session, careful patient selection and readiness to pursue surgical disconnection remain essential components of contemporary sDAVF management [1].

Despite extensive clinical experience, no universally accepted consensus exists regarding the optimal management strategy for sDAVFs. Both surgical and endovascular approaches have demonstrated favorable outcomes when appropriately selected, underscoring the importance of individualized, multidisciplinary decision-making. Increasingly, treatment algorithms emphasize an anatomy-driven approach that balances procedural risk with the likelihood of durable fistula obliteration.

In this study, we present our nine-year institutional experience in the multidisciplinary management of sDAVFs. Our treatment paradigm prioritizes endovascular embolization at the time of diagnostic angiography when feasible, reserving surgical intervention for cases in which embolization is unsuccessful, contraindicated, or complicated by recurrence. The objective of this study was to evaluate the safety and efficacy of this stepwise approach and to analyze clinical and angiographic outcomes in patients treated at our institution.

## 2. Materials and Methods

This retrospective study included patients diagnosed with sDAVFs who were treated between January 2015 and December 2023 at the Clinic of Neurosurgery, University Clinical Center of Serbia. Patients were identified through institutional medical records and imaging databases. Inclusion criteria comprised a confirmed diagnosis of sDAVF based on imaging, availability of complete clinical and radiological data, and a minimum of one follow-up assessment. Patients with alternative spinal vascular malformations or incomplete records were excluded.

The diagnosis of sDAVF was established using spinal MRI and DSA. MRI evaluation included T2-weighted sequences to assess intramedullary edema, identification of serpiginous perimedullary flow voids, and post-contrast sequences to evaluate abnormal vascular enhancement. DSA was performed to confirm the diagnosis, localize the fistulous point, identify arterial feeders and venous drainage patterns, and determine treatment feasibility.

Endovascular embolization was attempted as the first-line treatment strategy in all patients unless anatomical contraindications were present. Patients in whom angiography demonstrated a shared arterial origin with the anterior spinal artery (ASA) were considered unsuitable candidates for endovascular treatment because of the risk of inadvertent spinal cord ischemia and were referred directly for microsurgical disconnection. Consequently, such patients were not included in the present cohort, which specifically reflects our institutional endovascular-first treatment paradigm.

Technical success was defined as angiographic obliteration of the fistulous connection with penetration of the liquid embolic agent into the proximal draining vein. Particular emphasis was placed on occlusion of the foot of the draining vein, as failure to reach this segment is associated with fistula persistence and recurrence.

All embolization procedures were performed by a single experienced interventional neuroradiologist using a transarterial approach. A microcatheter was advanced as close as possible to the fistulous point under fluoroscopic guidance, with the aim of achieving complete occlusion of the arteriovenous shunt, including penetration into the proximal draining vein.

The following liquid embolic agents were utilized during the study period: Onyx 18 (Covidien, Irvine, CA, USA), PHIL 25 (MicroVention, Tustin, CA, USA), Menox™ 18 (Meril Life Sciences, Vapi, India), and Squid 12 (Balt Extrusion, Montmorency, France). Selection of the embolic agent was based on lesion angioarchitecture, microcatheter position, availability of materials, and operator preference. In selected cases, a combination of liquid embolic agents was employed to optimize penetration of the fistulous point and proximal draining vein.

Surgical intervention was reserved for patients in whom embolization was unsuccessful, incomplete, contraindicated, or complicated by recanalization. All surgical procedures were performed by two experienced neurosurgeons. Intraoperative localization of the fistula was guided by pre-procedural angiographic findings and intraoperative C-arm fluoroscopy. The presence of radiopaque embolic material from prior endovascular attempts facilitated precise intraoperative identification of the fistulous site. Laminotomy was performed at one or two levels adjacent to the angiographically identified fistula. Under microscopic visualization, the draining vein was identified, coagulated, and transected to achieve definitive disconnection of the arteriovenous shunt.

Clinical outcomes were assessed by comparing neurological status and presenting symptoms before and after treatment, with particular attention to motor function, sensory deficits, gait disturbances, and sphincter control. Neurological improvement was defined as partial or complete resolution of presenting symptoms or stabilization of previously progressive deficits. Radiological outcomes were evaluated using post-procedural DSA to assess fistula obliteration and follow-up MRI to evaluate regression of spinal cord edema and perimedullary venous congestion. Follow-up assessments were routinely conducted between 6 and 12 months after treatment.

Descriptive statistical analyses were performed using SPSS version 29.0 (IBM Corp., Armonk, NY, USA). Changes in pre- and post-treatment modified Aminoff and Logue Scale (mALS) scores were analyzed using the Wilcoxon signed-rank test. Statistical significance was defined as *p* < 0.05.

## 3. Results

A total of 15 patients were included in the study, comprising 14 males and 1 female, with a mean age of 57.8 years (range, 41–70 years). At presentation, two patients reported isolated chronic pain without objective neurological deficits, whereas the remaining 13 patients exhibited varying degrees of neurological impairment, including motor weakness, lower-extremity paresthesia, gait disturbance, and sphincter dysfunction. Incontinence was present in several patients at the time of diagnosis, reflecting advanced venous congestive myelopathy.

The anatomical distribution of sDAVFs spanned from the thoracic to the lumbar spine, with fistula levels ranging from Th5 to L5. Lower thoracic and upper lumbar locations were most frequently involved. In several cases, multilevel clinical symptoms were observed despite a single identifiable fistulous point, consistent with diffuse venous congestion affecting the spinal cord. Detailed demographic, clinical, and treatment characteristics as well as pre-operative and post-operative modified Aminoff and Logue’s Scale of the cohort are summarized in Table 1.

Endovascular embolization was attempted as the initial treatment modality in all patients using liquid embolic agents, including Onyx (Covidien, Irvine, CA, USA; *n* = 4), PHIL (MicroVention, Tustin, CA, USA; *n* = 4), Menox™ (Meril Life Sciences, Vapi, India; *n* = 4), and Squid (Balt Extrusion, Montmorency, France; *n* = 5). Combination use of embolic agents was employed in selected cases based on lesion angioarchitecture. Technical failure of embolization occurred in three patients (20%), with one failure observed for each embolic agent. In all three cases, embolization was deemed incomplete due to unfavorable angioarchitecture or insufficient penetration of the proximal draining vein, necessitating subsequent surgical disconnection.

Combined endovascular and surgical treatment was ultimately required in three patients, including those with previously failed embolization and patients with persistent or progressive symptoms following initial endovascular therapy. Surgical interruption of the draining vein was successfully achieved in all surgically treated cases, with no perioperative complications.

To further quantify neurological outcomes, pre- and post-treatment mALS subscores were compared using the Wilcoxon signed-rank test (Supplementary Table S1). A statistically significant improvement was observed in gait function following treatment (Z = −2.831, *p* = 0.005). Improvements in micturition and defecation scores were observed in selected patients; however, these changes did not reach statistical significance (both Z = −1.000, *p* = 0.317).

**Figure 1:**
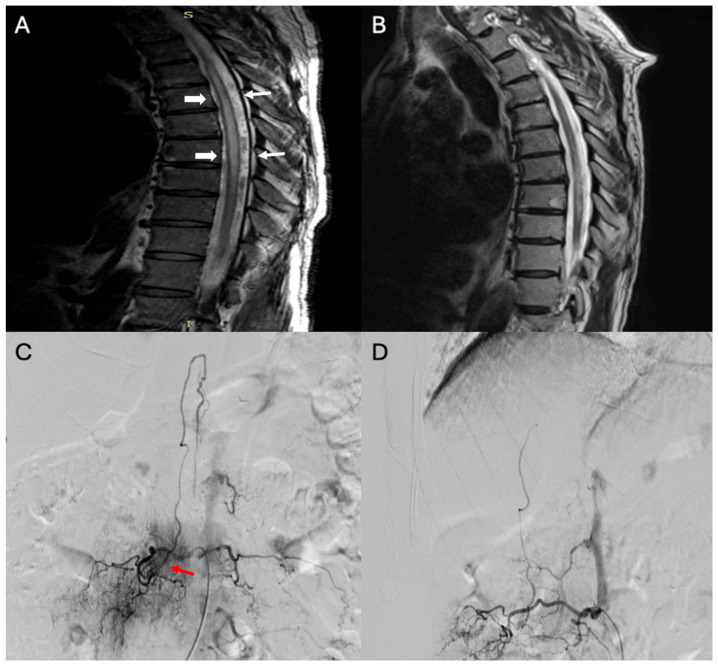
Representative imaging findings in a patient with spinal dural arteriovenous fistula (sDAVF). (**A**) Pre-treatment sagittal T2-weighted MRI showing tortuous and dilatated perimedullary blood vessels (small white arrows) and myelopathy (white arrows). (**B**) Follow-up sagittal T2-weighted MRI showing myelopathy in regression without dilatated perimedullary blood vessels in comparison with preprocedural MRI. (**C**) Pre-treatment digital subtraction angiography (DSA) with fistula point (small red arrow). (**D**) Post-treatment DSA confirming complete occlusion.

Complete angiographic occlusion of the fistula was confirmed in all patients at the conclusion of treatment (Figure 1). Figure 1 illustrates representative pre- and post-treatment MRI and DSA findings demonstrating typical imaging characteristics before intervention and complete occlusion of the arteriovenous shunt following definitive therapy.

From a clinical standpoint, neurological improvement was observed in 14 of 15 patients following definitive treatment. Improvements included reduction in pain, stabilization or improvement of motor function, and enhanced gait capacity. One patient with long-standing flaccid paraplegia did not demonstrate meaningful motor recovery, although a substantial reduction in pain symptoms was noted. No cases of neurological deterioration or treatment-related complications were observed during the follow-up period.

## 4. Discussion

In the present study, patients predominantly presented with a slowly progressive neurological course consistent with chronic venous congestion. The distribution of fistulas across the thoracolumbar spine and the frequent involvement of the conus medullaris align with previously reported hemodynamic patterns of venous hypertension [5].

Consistent with prior reports, patients in our cohort predominantly presented with a slowly progressive neurological course characterized by motor deficits, sensory disturbances, gait instability, and autonomic dysfunction [10,11]. The male predominance and mean age in the sixth decade observed in our series are consistent with established epidemiological patterns. Importantly, only a small subset of patients presented with isolated pain symptoms, whereas the majority exhibited advanced neurological impairment at the time of diagnosis, underscoring the nonspecific nature of early symptoms and the frequent delay in recognition of this entity [2]. Anatomically, fistulas were distributed across a broad thoracolumbar range (Th5–L5), reinforcing the concept that clinical and radiological manifestations may extend well beyond the anatomical level of the shunt due to diffuse venous congestion.

The primary therapeutic objective in sDAVF management is complete interruption of the arteriovenous shunt together with occlusion of the proximal draining vein. Proximal arterial occlusion alone is insufficient, as it fails to eliminate venous hypertension and is associated with high rates of fistula persistence or recurrence due to collateral recruitment [12]. Surgical disconnection has consistently demonstrated superior rates of definitive occlusion compared with endovascular embolization, with reported success rates exceeding 90% in multiple series [13,14]. A recent meta-analysis documented surgical occlusion rates approaching 98%, further reinforcing surgery as the most definitive modality for durable fistula cure [15]. Nevertheless, surgical intervention is inherently invasive and carries risks related to open spinal procedures, including cerebrospinal fluid leakage, wound complications, and potential neurological injury.

Endovascular embolization has therefore emerged as an attractive first-line strategy in selected patients, offering a minimally invasive approach that can be performed in conjunction with diagnostic angiography. In our cohort, endovascular embolization was attempted in all patients using contemporary liquid embolic agents, including Onyx (Covidien, Irvine, CA, USA), PHIL (MicroVention, Tustin, CA, USA), Squid (Balt Extrusion, Montmorency, France), and Menox™ (Meril Life Sciences, Vapi, India). Embolization was unsuccessful in three cases, each involving a different embolic agent, suggesting that treatment failure was more closely related to lesion angioarchitecture and venous drainage patterns than to the specific embolic material employed. In these patients, subsequent surgical disconnection resulted in definitive fistula obliteration, highlighting the importance of maintaining surgical readiness as part of a comprehensive treatment strategy.

Although the small cohort size precludes formal statistical comparison between embolic agents, a descriptive analysis did not reveal clear superiority of any single material. Embolization failure occurred across different agents, suggesting that procedural outcome was more strongly influenced by lesion angioarchitecture and venous drainage patterns than by intrinsic properties of the embolic material. All agents demonstrated satisfactory handling characteristics and radiographic visibility in cases of successful occlusion. These observations, while limited by sample size, are consistent with prior reports indicating broadly comparable efficacy among contemporary EVOH-based embolic agents [9].

Neurological outcomes in our series were favorable overall. With the exception of one patient who presented with advanced paraplegia, all patients demonstrated neurological improvement following treatment, including reductions in pain, improvements in motor function, and stabilization or partial recovery of gait. The lack of meaningful motor recovery in the patient with persistent paraplegia likely reflects irreversible spinal cord damage due to prolonged venous congestion prior to intervention, consistent with the established prognostic importance of symptom duration and baseline neurological status [16,17]. Notably, even in this case, substantial pain relief was achieved, indicating that symptomatic benefit may still be attainable despite limited functional recovery [16,17]. In rare circumstances, particularly among individuals with long-standing deficits, clinical deterioration may occur despite complete angiographic cure, likely reflecting irreversible myelopathic injury prior to treatment [18]. Accordingly, recurrence or recanalization should be considered in patients who initially improve but subsequently plateau or deteriorate clinically, and repeat imaging/angiographic evaluation may be warranted in such scenarios [19].

Notably, our cohort consisted exclusively of symptomatic patients at the time of diagnosis. The applicability of this stepwise treatment strategy in asymptomatic or incidentally detected sDAVFs remains uncertain and warrants further prospective investigation, particularly in the context of earlier intervention prior to irreversible myelopathic injury.

Within this symptomatic population, our findings further support a multidisciplinary, stepwise treatment paradigm in which endovascular embolization serves as an initial therapeutic and diagnostic modality, with surgical disconnection reserved for refractory, anatomically unfavorable, or recurrent cases. Importantly, prior embolization facilitated intraoperative positioning assistance in patients requiring subsequent surgery. The radiopaque embolic cast functioned as a reliable anatomical landmark, facilitating precise localization of the fistulous point and proximal draining vein. In our experience, this reduced the need for extensive intradural exploration and may have contributed to shorter operative time and lower procedural morbidity.

This synergistic interaction between endovascular and surgical strategies represents a tangible procedural advantage of an endovascular-first approach, even when embolization does not achieve definitive occlusion. These multidisciplinary principles align with evolving expert consensus on the integrated management of spinal vascular malformations, as detailed in recent comprehensive reviews, further supporting a combined endovascular–surgical strategy [20]. Rather than reflecting treatment failure, initial embolization may therefore enhance the safety and efficiency of subsequent microsurgical disconnection.

Taken together, our experience demonstrates that an endovascular-first strategy, integrated within a multidisciplinary framework and coupled with timely surgical intervention when necessary, can achieve favorable clinical outcomes with an acceptable safety profile. These findings reinforce the importance of early diagnosis, careful anatomical assessment, and flexible treatment algorithms in optimizing outcomes for patients with sDAVFs.

### Study Limitations

Several limitations of this study warrant consideration. First, the retrospective, single-center design inherently limits the generalizability of our findings and introduces the potential for selection and referral bias. Given the rarity of sDAVFs, the sample size was limited to 15 patients, which restricts statistical power and precluded robust subgroup analyses or multivariable modeling to identify independent predictors of clinical or angiographic outcomes. The small cohort size also limits the external validity of our findings, as institutional experience, operator expertise, and case selection patterns may not be fully representative of broader clinical practice. Therefore, while our results support the feasibility and safety of a stepwise multidisciplinary approach, caution should be exercised when generalizing these conclusions to other centers or patient populations.

Second, the heterogeneity of embolic agents used reflects real-world clinical practice but complicates direct comparison of treatment efficacy across agents. Although embolization failures occurred across different materials, definitive conclusions regarding the relative performance of specific liquid embolic agents cannot be drawn from this cohort. Similarly, treatment allocation was not randomized and was influenced by lesion anatomy, operator judgment, and temporal availability of embolic materials.

Third, neurological outcomes were assessed clinically without the use of standardized functional scoring instruments across all patients, limiting the precision of outcome quantification. While neurological improvement was observed in the majority of patients, subtle changes in functional status or quality of life may not have been fully captured.

Fourth, follow-up duration varied among patients, and long-term angiographic surveillance was not uniformly available. As late recanalization or recurrence has been reported in sDAVFs, particularly following endovascular treatment, the true durability of fistula occlusion in this cohort may be underestimated.

Finally, although our study highlights the advantages of a multidisciplinary, stepwise treatment algorithm, it does not directly compare this approach with primary surgical management. Prospective, multicenter studies with standardized outcome measures and longer follow-up are needed to further refine treatment algorithms and optimize patient selection for endovascular versus surgical intervention and make findings more generalizable.

## 5. Conclusions

Spinal dural arteriovenous fistula (sDAVF) is a rare but highly treatable cause of otherwise progressive paraplegia. Neuroradiologists play a pivotal role not only in the detection of these lesions but also in guiding their management. Although neurological symptoms are frequently nonspecific, the MRI triad of spinal cord edema, dilated perimedullary vessels, and contrast enhancement, particularly in elderly male patients, should raise a strong clinical suspicion, with selective digital subtraction angiography remaining the diagnostic gold standard.

Our institutional treatment algorithm demonstrates that a stepwise, multidisciplinary approach, prioritizing endovascular embolization and reserving surgical intervention for refractory or recurrent cases, is both safe and effective. Most patients experienced neurological improvement, highlighting the value of an integrated management strategy in optimizing clinical outcomes.

## Figures and Tables

**Table 1:** Study Cohort Characteristics.

N	Age	Gender	Level	Clinical Presentation	mALS	Embolization/Surgery	Outcome	Postoperative Outcome	Postoperative mALS
1	49	M	L1	Left foot paresthesia Popliteal fossa pain No motor deficit	G 0 M 0 D 0	Onyx 18	Complete occlusion	No motor deficit	G 0 M 0 D 0
2	41	M	Th5	Progressive flaccid paraplegia MMT 0/5 Sensory level Th5 No sphincter control	G 5 M 3 D 3	Onyx 18	Complete occlusion	Paraparesis 3/5 Sensory recovery No sphincter control	G 3 M 3 D 3
3	45	M	Th10	Left lower extremity weakness MMT 3/5 Ataxic gait	G 4 M 0 D 0	Squid 12	Complete occlusion	MMT 4/5 on the left lower extremity	G 1 M 0 D 0
4	65	M	L1–L2	Back pain Feet paresthesia, cold feet m. iliopsoas and hamstrings MMT 4/5 Hypotrophy left hamstring Walking difficulties Urinary urgency	G 1 M 1 D 0	Squid 12	Complete occlusion	No change in motor deficit Urinary urgency	G 1 M 1 D 0
5	57	F	Th7	Left m. tibialis anterior MMT 3/5 Radicular hypesthesia left S1, both lower extremity paresthesia No sphincter control	G 4 M 3 D 3	Onyx 18 Squid 12 Surgery	Complete occlusion	Left m. tibialis anterior MMT 4/5 No sphincter control	G 1 M 3 D 3
6	60	M	L5	Spastic paraparesis, MMT 2/5 Walking difficulties Urinary urgency	G 4 M 1 D 0	Phil 25	Complete occlusion	Paraparesis MMT 3/5 Sphincter control	G 3 M 1 D 0
7	67	M	Th10	Back pain Flaccid paraparesis, MMT proximally 3/5, distally 2/5 Walking difficulties	G 4 M 0 D 0	Phil 25 Surgery	Complete occlusion	No motor deficit	G 0 M 0 D 0
8	55	M	Th11–Th12	Spastic paraparesis, MMT left 4/5, right 3/5 Lower extremity paresthesia Walking difficulties	G 1 M 0 D 0	Onyx 18	Complete occlusion	Paraparesis MMT 4/5	G 1 M 0 D 0
9	52	M	Th6–Th12	Progressive spastic paraparesis, MMT 4/5 Walking difficulties	G 1 M 0 D 0	Onyx 18	Complete occlusion	No motor deficit	G 0 M 0 D 0
10	69	M	Th9–Th10	Progressive spastic paraparesis, MMT 4/5 Walking difficulties	G 1 M 0 D 0	Phil 25	Complete occlusion	No motor deficit	G 0 M 0 D 0
11	70	M	L1	Progressive flaccid paraparesis, MMT 0/5 Sensory level Th10 Lower extremity paresthesia Unable to walk Urinary urgency	G 5 M 1 D 0	Phil 25	Complete occlusion	Flaccid paraparesis, MMT 0/5 Sensory recovery Urinary urgency	G 5 M 1 D 0
12	54	M	Th11	Lower extremity hypotonia, no motor deficit Left foot paresthesia	G 0 M 0 D 0	Menox 18	Complete occlusion	No motor deficit Occasional left foot paresthesia	G 0 M 0 D 0
13	60	M	Th6	Progressive spastic paraparesis MMT 3/5 Walking difficulties	G 3 M 0 D 0	Squid 12 Surgery	Complete occlusion	Paraparesis MMT 4/5	G 1 M 0 D 0
14	59	M	Th6–Th12	Progressive spastic paraparesis, MMT 4/5 right, 3/5 left Walking difficulties	G 3 M 0 D 0	Menox 18	Complete occlusion	Paraparesis MMT 4/5	G 1 M 0 D 0
15	64	M	L2	Back pain Spastic paraparesis MMT 3/5 Walking difficulties No sphincter control	G 3 M 3 D 3	Menox 18 Squid 12	Complete occlusion	No motor deficit	G 1 M 1 D 1

mALS—modified Aminoff and Logue’s Scale; G—gait; M—micturition; D—defecation; MMT—manual motor testing.

## Data Availability

Data available upon request from the authors due to privacy reasons.

## Supplementary Materials

The following supporting information can be downloaded at: https://www.mdpi.com/article/10.3390/neurolint18070137/s1, Table S1: Comparison of pre- and post-treatment modified Aminoff and Logue Scale (mALS) subscores using the Wilcoxon signed-rank test.
