# Supplementary material for: Multidisciplinary Management of Spinal Dural Arteriovenous Fistulas Using an Endovascular-First Treatment Strategy: A Nine-Year Single-Center Experience
Source: Neurol Int. 2026 Jul 16;18(7):137. doi: 10.3390/neurolint18070137 (PMC13415202; doi:10.3390/neurolint18070137)
Supplement: Supplementary file 1 [file neurolint-18-00137-s001.zip › neurolint-4338486-supplementary.pdf]

Supplementary Table S1. Comparison of pre- and post-treatment modified Aminoff and Logue Scale (mALS) subscores using the Wilcoxon signed-rank test

mALS domain	Pre-treatment Median (IQR)	Post-treatment Median (IQR)	Z	p-value
Gait	3 (1–4)	1 (0–1)	-2.831	0.005
Micturition	0 (0–1)	0 (0–1)	-1.000	0.317
Defecation	0 (0–0)	0 (0–0)	-1.000	0.317
